# AEG-1/MTDH-activated autophagy enhances human malignant glioma susceptibility to TGF-β1-triggered epithelial-mesenchymal transition

**DOI:** 10.18632/oncotarget.7536

**Published:** 2016-02-20

**Authors:** Meijuan Zou, Wei Zhu, Li Wang, Lei Shi, Rui Gao, Yingwei Ou, Xuguan Chen, Zhongchang Wang, Aiqin Jiang, Kunmei Liu, Ming Xiao, Ping Ni, Dandan Wu, Wenping He, Geng Sun, Ping Li, Sulan Zhai, Xuerong Wang, Gang Hu

**Affiliations:** ^1^ Department of Pharmacology, School of Basic Medical Sciences, Nanjing Medical University, Nanjing 210029, China; ^2^ Department of Oncology, First Affiliated Hospital of Nanjing Medical University, Nanjing 210029, China; ^3^ Department of Breast Surgery, First Affiliated Hospital of Nanjing Medical University, Nanjing 210029, China; ^4^ State Key Laboratory of Pharmaceutical Biotechnology, Nanjing University, Nanjing 210093, China; ^5^ Medical School of Nanjing University, Nanjing 210093, China; ^6^ Ningxia Key Laboratory of Cerebrocranial Diseases, School of Laboratory Medicine, Ningxia Medical University, Yinchuan 750004, China; ^7^ Department of Anatomy, School of Basic Medical Sciences, Nanjing Medical University, Nanjing 210029, China

**Keywords:** transforming growth factor-β1, astrocyte elevated gene-1, protective autophagy, epithelial mesenchymal transition, malignant glioma invasion

## Abstract

Autophagy is a tightly regulated process activated in response to metabolic stress and other microenvironmental changes. Astrocyte elevated gene 1 (AEG-1) reportedly induces protective autophagy. Our results indicate that AEG-1 also enhances the susceptibility of malignant glioma cells to TGF-β1-triggered epithelial-mesenchymal transition (EMT) through induction of autophagy. TGF-β1 induced autophagy and activated AEG-1 via Smad2/3 phosphorylation in malignant glioma cells. Also increased was oncogene cyclin D1 and EMT markers, which promoted tumor progression. Inhibition of autophagy using siRNA-BECN1 and siRNA-AEG-1 suppressed EMT. In tumor samples from patients with malignant glioma, immunohistochemical assays showed that expression levels of TGF-β1, AEG-1, and markers of autophagy and EMT, all gradually increase with glioblastoma progression. *In vivo* siRNA-AEG-1 administration to rats implanted with C6 glioma cells inhibited tumor growth and increased the incidence of apoptosis among tumor cells. These findings shed light on the mechanisms underlying the invasiveness and progression of malignant gliomas.

## INTRODUCTION

Malignant glioma originates in the cerebral glia of brain tissue. No obvious boundary between glioma and brain tissue exists due to its invasive growth [1]. Radiotherapy or chemotherapy following surgery as combination therapy is crucial [2]. Elucidating the molecular mechanism and signaling molecules of malignant glioma invasion and progression can provide a theoretical basis for the clinical diagnosis and treatment of malignant gliomas. This will not only increase the survival rate of patients but also provide new strategies for the development of selective and potent resistant malignant glioma drugs.

AEG-1 has been first identified in research on Alzheimer's disease or migraines caused by HIV virus and then found at high expression levels in a variety of tumors, participating in tumor occurrence and development [3–5]. AEG-1 is localized in the perinuclear region and in the endoplasmic reticulum [6]. In recent years, research has shown that AEG-1 is a downstream molecule of Ha-ras and cyclin D1, which can activate the PI3K-Akt, NF-κB and Wnt signaling pathways, which are closely associated with autophagy in cancer cells [7–9]. AEG-1 has been reported to reduce cellular metabolism and to activate adenylate kinase by reducing the ATP/AMP ratio, which induced autophagy via inhibition of AMPK/mTOR signaling. Such protective autophagy can promote cell survival in the tumor microenvironment and may directly contribute to tumor cell transformation [10].

Tumor cells must adapt to different survival pressures during the complex and multistep biological process of tumor invasion and progression. Autophagy (here referring to macroautophagy) is a highly conserved process of cell self-eating [11]. One of its most important functions is to remove excess, damaged, cancerous or microbially infected cells to maintain stability in the internal environment [12]. Autophagy not only can be activated to adapt to metabolic stress and micro-environmental changes but also may promote cancer cell invasion and metastasis, such as epithelial-mesenchymal transition (EMT) and the inflammatory environment [13].

EMT refers to a transformation whereby epithelial cells with polarity become freely moving mesenchymal cells in the intercellular space under specific physiological and pathological situations [14]. EMT has become one of the most exciting fields in cancer biology especially in cancer cell invasion, metastasis and drug resistance. Recent studies have shown that autophagy activation in tumor cells suppressed Wnt signaling pathway via the degradation of cytosolic and disheveled proteins, which participate in EMT process [15]. Here, we report that autophagy coordinately sensitizes EMT and promotes malignant glioma invasion and progression.

Transforming growth factor beta (TGF-β) super-family is polypeptide cytokines that are extensively involved in the regulation of cell biological processes such as proliferation, differentiation, invasion and migration [16]. TGF-β1 is able to stimulate molecules on signal transduction, to promote the accumulation of extracellular matrix proteins, and to induce EMT [17]. TGF-β1, with high expression and secretion levels in glioma, may be the primary factor that promotes brain glioma invasion and progression. TGF-β1 is a key mediator of EMT and is correlated with the expression of several epithelial cell recognition and organizational proteins, including E-cadherin and N-cadherin [18]. Furthermore, evidence has shown that both the Smad-dependent and the Smad-independent TGF-β1-activated EMT processes are involved in the regulation of autophagy activation [19].

However, the correlation between autophagy activation and EMT mechanisms of TGF-β1 has not been elucidated. Furthermore, AEG-1 and EMT have not been studied in the context of glioma. Therefore, our objective is to study the mechanism underlying the variation in the tumor environment and the consequent progression of malignant glioma. We hypothesized that AEG-1 might act beneficially during autophagy activation. This hypothesis has been validated by our experiments. Our results demonstrated that autophagy flux is activated within 3 hours and accompanied by the up-regulation of AEG-1 in malignant glioma cells following TGF-β1 (5 ng/ml) treatment for 1, 3, 6, 12 and 24 hours. The expression of the oncogene cyclin D1 and EMT markers increased with AEG-1, suggesting that this biological process may promote tumor progression. Autophagy inhibition by siBECN1 and siAEG-1 pretreatment reversed the expression levels of the above markers. Thus, our data support the hypothesis that AEG-1 that stimulated glioma cell autophagy and enhanced EMT and progression of gliomas.

## RESULTS

### AEG-1/MTDH is activated during TGF-β1-triggered EMT in malignant glioma cells

**Figure 1:**
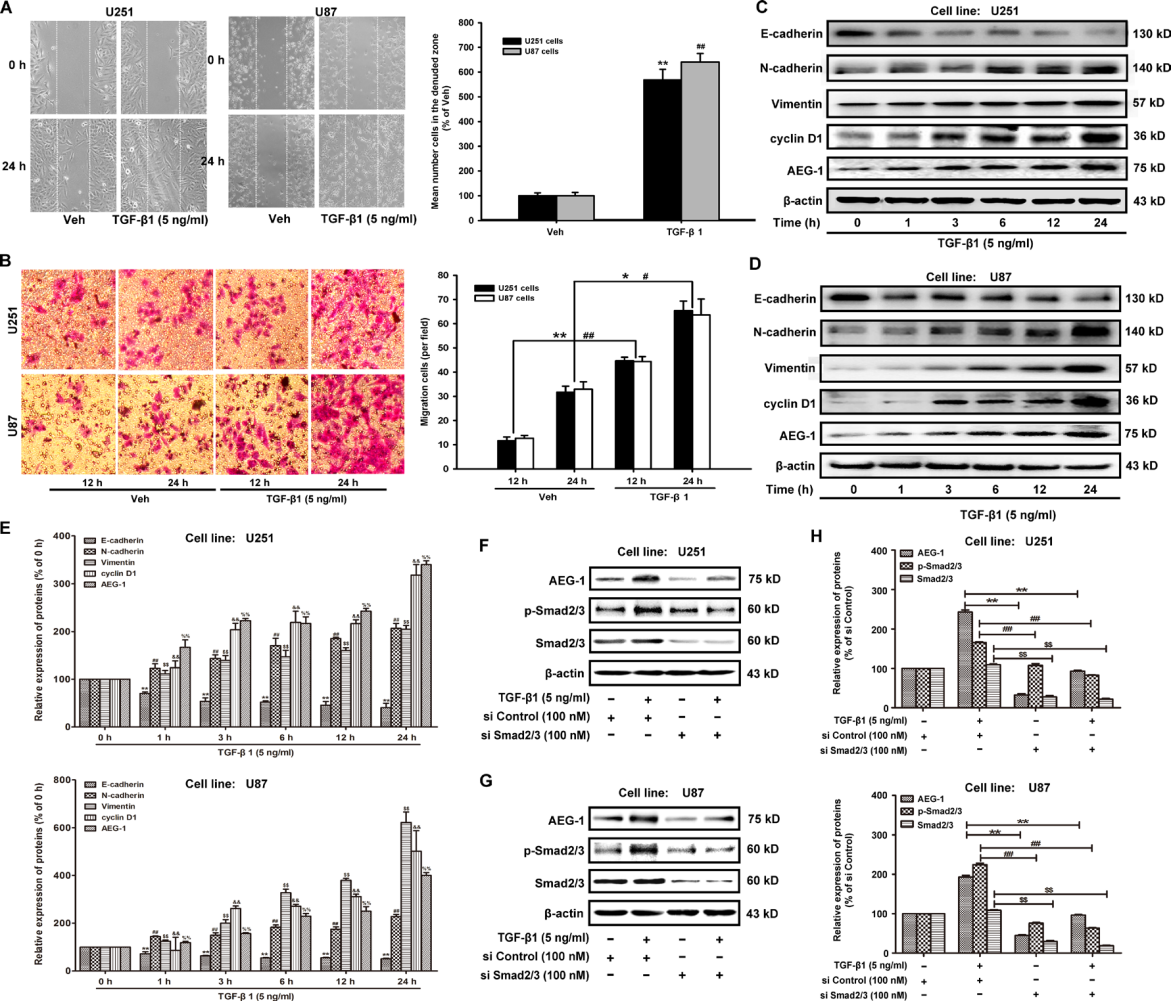
TGF-β1 increases AEG-1 expression and induces EMT and invasion in malignant glioma cells (**A**) U251 and U87 cells are plated in 6-well plates, and a confluent monolayer is wounded using a sterile micropipette tip. Then, the cells are treated with TGF-β1 (5 ng/ml) for 24 hours after wounding. The migrating cells in the denuded zone are assessed using an inverted microscope. White dotted lines indicate the wound edge. Image magnification: 100 ×. (**B**) After U251 and U87 cells are cultured in the presence of TGF-β1 (5 ng/ml) or vehicle for 12 or 24 hours, respectively, the cells are seeded in the upper portion of a Transwell coated with Matrigel. After the cells are incubated for 24 hours, the downward side of the membrane is stained with crystal violet solution. Image magnification: 200 ×. The invading cells are counted in five random fields for each treatment. (**C** and **D**) The protein expression of E-cadherin, N-cadherin, vimentin, cyclin D1 and AEG-1 in U251 and U87 cells treated with TGF-β1 (5 ng/ml). β-actin is used as internal controls to ascertain equal loading. (**E**) Representative quantitative data of densitometric analyses. The ratios of proteins to β-actin are present as mean ± SD, *n* = 3, *, ^#^, ^$^, *P* < 0.05 and **, ^##^, ^$$^, *P* < 0.01 compared to vehicle-treated cells or si Control-transfected cells. (**F** and **G**) TGF-β1 induces the expression of AEG-1 via activation of Sma2/3 signaling in malignant brain glioma cell line U87 and U251. Western blot analysis shows the effect of TGF-β1 treatment of cells transfected with si Smad2/3 or si Control on AEG-1 expression. (**H**) Representative quantitative data of densitometric analyses. Data represent the mean ± SD of three different experiments. *, ^#^, ^$^, *P* < 0.05 and **, ^##^, ^$$^, *P* < 0.01 compared to vehicle-treated cells or si Control-transfected cells.

TGF-β1 has been reported to be the most important regulator of EMT and cell migration in human lung tumor cells [20]. In our present study, the motility and invasion abilities of U251 and U87 cells treated with TGF-β1 (5 ng/ml), as measured by wound healing and transwell invasion assays. TGF-β1 induces EMT in human malignant glioma cells, as characterized by morphological changes from an epithelial shape to an elongated shape (Figure 1A). The migration cells are increased by TGF-β1 from 11.67 ± 1.53 to 44.67 ± 1.53 (U251); 12.67 ± 1.15 to 44.33 ± 2.08 (U87) by 12 hours. The migration cells are increased by TGF-β1 from 31.67 ± 2.52 to 65.33 ± 4.04 (U251); 33.00 ± 3.00 to 63.67 ± 6.51 (U87) by 24 hours (Figure 1B).

To identify these effects at the molecular level, western blot analyses are performed to determine the expression of AEG-1/MTDH and EMT hallmarks. First, the expression of AEG-1 and its downstream cyclin D1 are increased in both U251 and U87 malignant glioma cells by treatment with TGF-β1 (5 ng/ml) for 1, 3, 6, 12 and 24 hours (Figure 1C, 1D and 1E). The expression of the epithelial marker E-cadherin is strongly decreased when U251 and U87 malignant glioma cells are treated with TGF-β1 (Figure 1C). Conversely, the expression of mesenchymal markers such as N-cadherin and vimentin is enhanced by TGF-β1 treatment (Figure 1C). Relative gene expression of E-cadherin, N-cadherin, Vimentin and Snail was detected by real-time reverse transcription-polymerase chain reaction (RT-PCR) assays. TGF-β1 treatment even reduced E-cadherin expression and increased N-cadherin, Vimentin and Snail expression (Supplementary Figure S1). Finally, the expression of AEG-1 changes the most compared with the expression of N-cadherin and vimentin during EMT in U251 and U87 malignant glioma cells. These data suggest that a positive correlation exists between the up-regulation of AEG-1 and mesenchymal hallmarks in malignant glioma cells. In addition, as shown in Figure 1E and 1F, TGF-β1 treatment induces the expression of AEG-1 via activation of Smad2/3 signaling as the ratio of p-Smad2/3: 165.30 ± 1.93 (U251) and 224.24 ± 4.30 (U87), % of si Control respectively (Figure 1H). Knockdown of Smad2/3 by siRNA-Smad2/3 rescues the constitutively active Smad2/3 and decreases the expression of AEG-1 from 242.57 ± 5.70 to 32.19 ± 3.29 and 92.94 ± 1.92 (U251); from 193.00 ± 4.15 to 44.57 ± 2.20 and 96.43 ± 1.54 (U87), % of si Control respectively (Figure 1H).

### Autophagy is involved in TGF-β1-triggered EMT in malignant glioma cells

**Figure 2:**
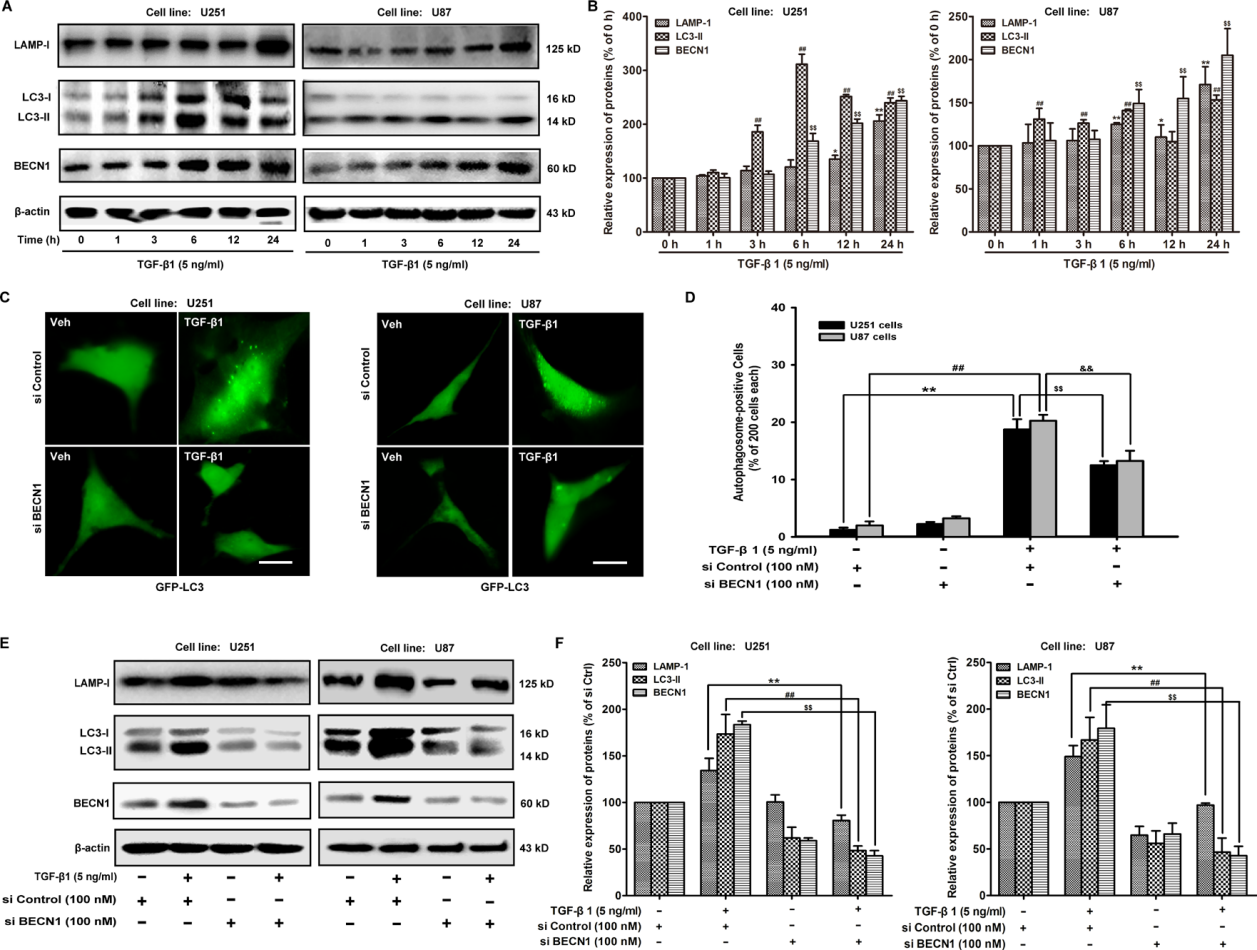
TGF-β1 induces autophagy in malignant glioma cells (**A** and **B**) Effect of TGF-β1 on MAP-LC3 lipidation and BECN1, LAMP-1 expression levels in cells are analyzed by western blot. U251 and U87 cells are treated with TGF-β1 (5 ng/ml) for the indicated times. β-actin is used as internal controls to ascertain equal loading. (**C**) U251 and U87 cells are transfected with pcDNA3.1-GFP-MAP-LC3 and si BECN1 or si Control using Lipofectamine^™^ 2000 for 24 hours and then treated with TGF-β1 (5 ng/ml) and or vehicle for another 24 hours. GFP-MAP-LC3-labeled cells are monitored by fluorescence microscopy at a 600 × magnification. Scale bar: 200 μm. (**D**) Quantification of the percentage of cells with focal GFP-MAP-LC3 at the indicated times after treatment. Error bars correspond to SD of three independent experiments (200 cells were counted per experiment). (**E** and **F**) Western blot analysis shows the effect of TGF-β1 treatment of cells transfected with si BECN1 or si Control on MAP-LC3 lipidation and LAMP-1 expression. **, ^##^*P* < 0.01 compared to vehicle-treated cells. ^$$^, ^&&^*P* < 0.01 compared to si Control-transfected cells.

Next, we determine whether TGF-β1 could induce autophagy during EMT in malignant glioma cells. The conversion of the soluble form of MAP-LC3 (LC3-I) to the lipidated and autophagosome-associated form (LC3-II) is considered as one of the hallmarks of autophagy [21]; thus, we evaluate the expression of Beclin 1 (BECN1) and the appearance of LC3-II in both U251 and U87 malignant glioma cells following treatment with TGF-β1 (5 ng/ml) for 1, 3, 6, 12 and 24 hours (Figure 2A and 2B). We also evaluate well-known lysosomal markers, such as the outer lysosomal protein LAMP-1, whose expression increased upon TGF-β1 treatment (Figure 2A and 2B). These results indicate that autophagy is a key function of the lysosomal compartment.

To explore whether BECN1 is involved in TGF-β1-induced malignant glioma cell autophagy, we examine the effect of siRNA BECN1 on malignant glioma cell autophagy. We observe a decrease in the occurrence of GFP-LC3-positive dots, as well as the down-regulation of LC3-II and LAMP-1 in TGF-β1-stimulated cells (Figure 2C). Because MAP-LC3 is an essential hallmark for autophagosome formation, we further study the TGF-β1-triggered intracellular localization of MAP-LC3 in the autophagosome by transiently transfecting U251 and U87 malignant glioma cells with a plasmid-expressing GFP fused with LC-3 (pcDNA3.1-GFP-LC3) followed by TGF-β1 treatment for 24 hours. In si Control-treated cells, GFP-LC3 is observed predominantly as a diffuse green fluorescence in the cytoplasm. The autophagosome-positive cells are counted as 1.25 ± 0.35 (U251) and 2.00 ± 0.71 (U87), % of 200 cells. However, characteristic punctate GFP-LC3-positive cells with TGF-β1 treatment are detected as 18.75 ± 1.77 (U251) and 20.25 ± 1.06 (U87), % of 200 cells. Si BECN1 decreases the autophagosome-positive cells as 12.50 ± 0.71 (U251) and 13.25 ± 1.77 (U87), % of 200 cells, indicating the recruitment of GFP-LC3 during autophagosome formation (Figure 2D). The expression of LC3-II and LAMP-1 in malignant glioma cells are rescued by si BECN1 following treatment with TGF-β1 (5 ng/ml) for 24 hours. The ratio of LC3-II and LAMP-1 expression are decreased by si BECN1from 173.46 ± 21.01 to 48.38 ± 4.89 and 134.26 ± 13.13 to 80.64 ± 5.77 (U251); 166.91 ± 24.19 to 46.54 ± 15.11 and 149.01 ± 11.84 to 96.93 ± 2.13 (U87), % of si Control respectively (Figure 2E and 2F).

**Figure 3:**
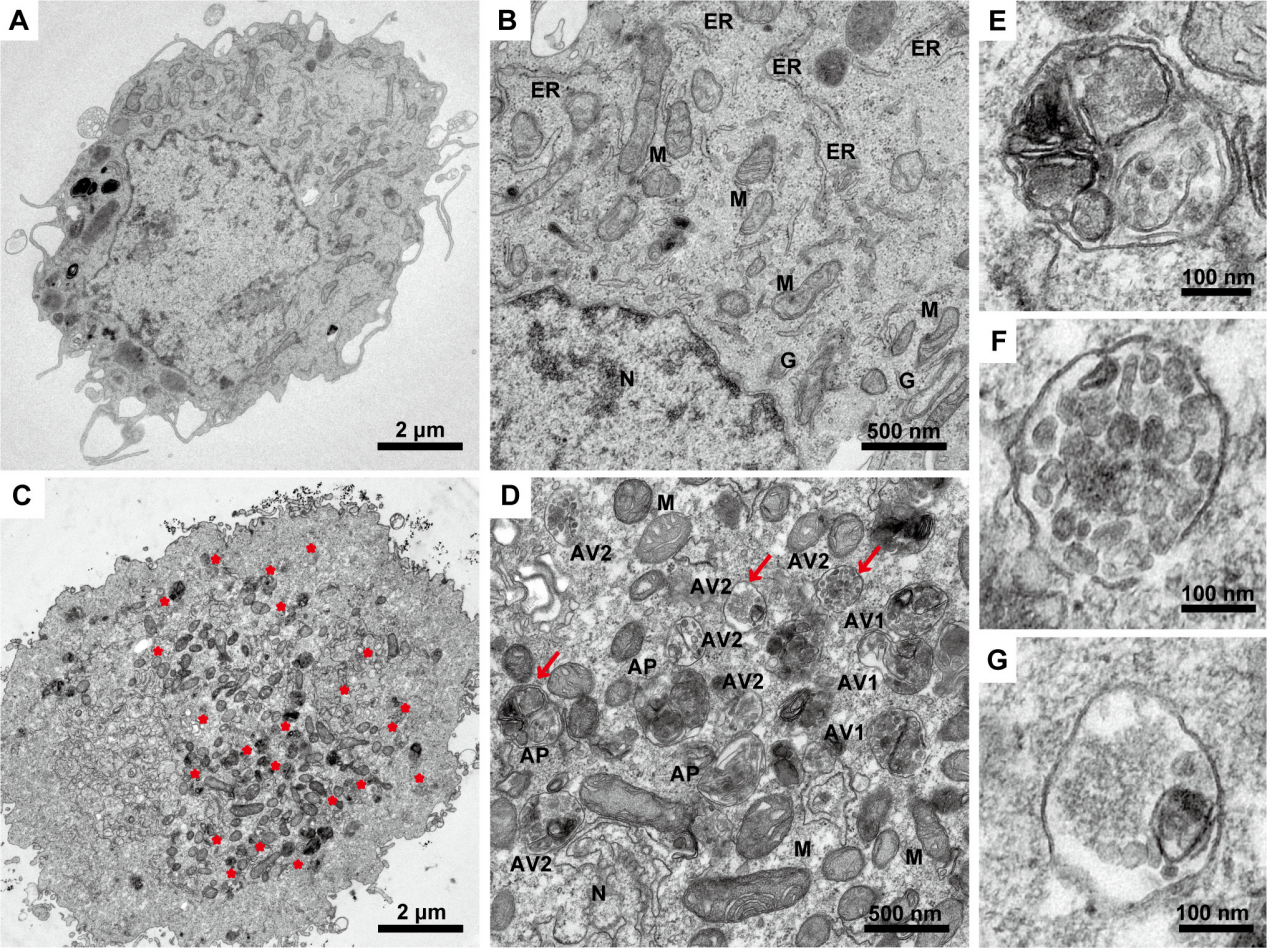
Transmission electron microscopy images of U87 malignant glioma cells following TGF-β1 treatment The cells are treated with TGF-β1 (5 ng/ml) or vehicle for 24 hours, fixed immediately in 1% glutaraldehyde and post-fixed in 2% osmium tetroxide. The cell pellets or sections are embedded in epon resin. Representative areas are chosen for ultrathin sectioning, and transmission electron microscopy is performed using a Tecnai G2 Spirit Bio TWIN (FEI Co., Netherlands) at 120 kV. (**A** and **B**) U87 cells treated with vehicle as a control with normal nuclear and organelles. N, nuclear; ER, endoplasmic reticulum; M, mitochondrion; G, Golgi body. Scale bars: 2 μm (A) and 500 nm (B). (**C** and **D**) U87 cells are treated with TGF-β1 (5 ng/ml). Red asterisks, autophagy area; red arrows, representative autophagic structures; N, nuclear; M, mitochondrion; AP, initial autophagic phagophore (isolation membrane) (**E**); AV1, autophagosomes as autophagic vacuoles 1 (**F**); AV2, autolysosomes as autophagic vacuoles 2 (**G**). Scale bars: 2 μm (C), 500 nm (D) and 100 nm (E, F, and G).

As a gold standard to gain insight into the morphological changes induced in malignant glioma cells upon TGF-β1 administration, we perform transmission electron microscopy analysis of U87 cells. Notably, TEM images reveal an accumulation of numerous large autophagy-related structures in the cytoplasm of TGF-β1-treated cells (Figure 3C and 3D) compared to vehicle-treated cells (Figure 3A and 3B). Because autophagy is activated, double isolation membrane structures (autophagic phagophore, AP) from the rough endoplasmic reticulum without ribosomes wrapped around the degradation organelles, proteins and other ingredients to form autophagosomes (autophagic vacuole 1, AV1) and converted into autolysosomes (autophagic vacuole 2, AV2) after fusion with lysosomes (Figure 3E, 3F and 3G).

### AEG-1/MTDH overexpression enhances TGF-β1-induced autophagy and EMT in malignant glioma cells

**Figure 4:**
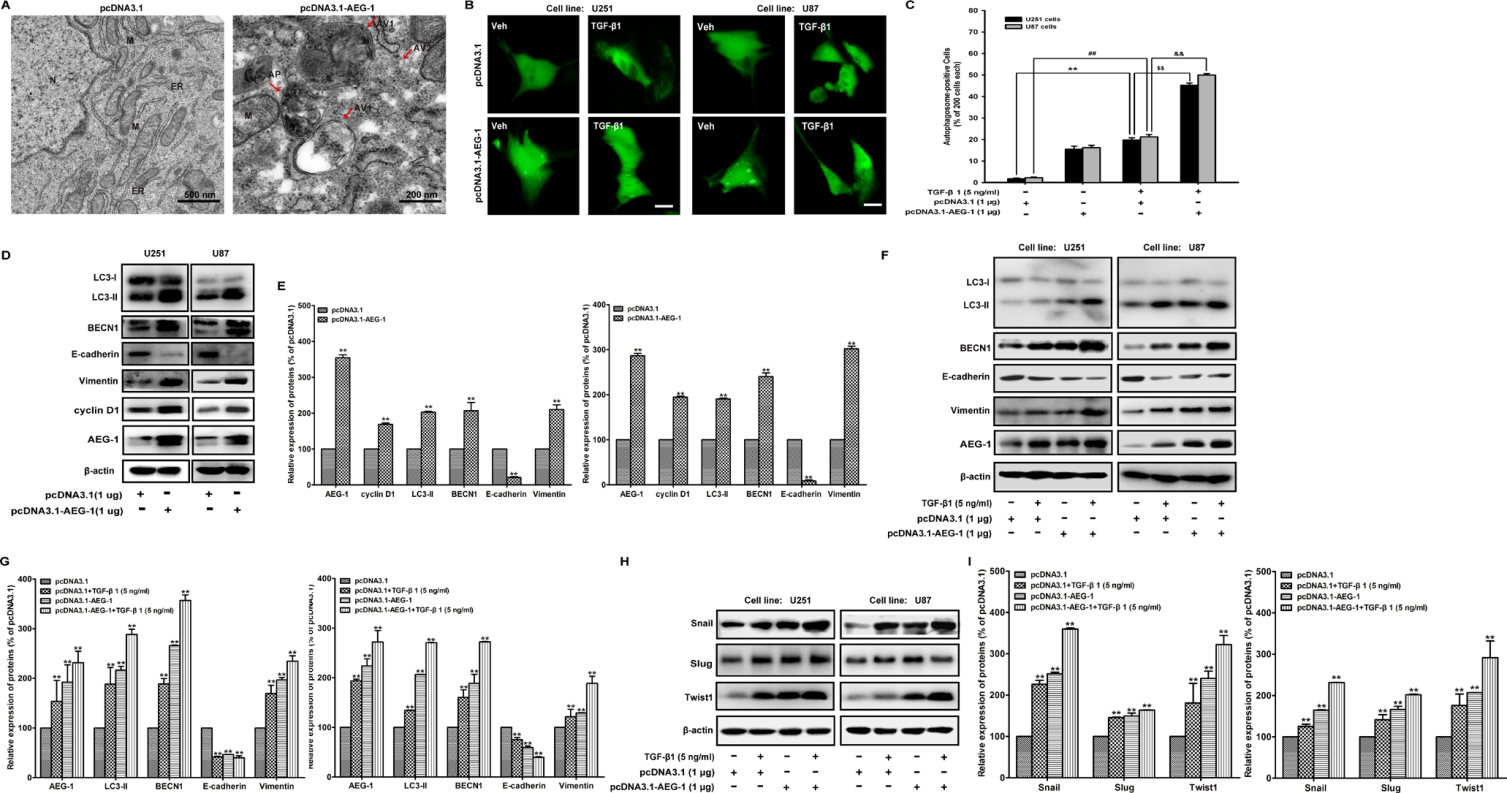
AEG-1 overexpression enhances TGF-β1-induced autophagy and EMT in malignant glioma cells (**A**) Transmission electron microscopy images of U87 malignant glioma cells after transfection with pcDNA3.1-AEG-1/pcDNA3.1. N, nuclear; ER, endoplasmic reticulum; M, mitochondrion; AP, initial autophagic phagophore (isolation membrane); AV1, autophagosomes as autophagic vacuoles 1; AV2, autolysosomes as autophagic vacuoles 2. Scale bars: 500 nm (upper) and 200 nm (lower). (**B**) U251 and U87 cells are transfected with pcDNA3.1-GFP-MAP-LC3 and pcDNA3.1-AEG-1/pcDNA3.1 using Lipofectamine™ 2000 for 24 hours and then treated with TGF-β1 (5 ng/ml) or vehicle for another 24 hours. GFP-MAP-LC3-labeled cells are monitored by fluorescence microscopy at 600 × magnification. Scale bar: 200 μm. (**C**) Quantification of the percentage of cells with focal GFP-MAP-LC3 at the indicated times after treatment. Error bars correspond to SD of three independent experiments (200 cells were counted per experiment). **, ^##^*P* < 0.01 compared to vehicle-treated cells. ^$$^, ^&&^*P* < 0.01 compared to pcDNA3.1-transfected cells. (**D** and **E**) U251 and U87 cells are transfected with pcDNA3.1-AEG-1/pcDNA3.1 using Lipofectamine™ 2000 for 24 hours, the expression of AEG-1 and cyclin D1 and MAP-LC3 lipidation and BECN1, E-cadherin, and vimentin are analyzed by western blot. β-actin is used as internal controls to ascertain equal loading. (**F** and **H**) U251 and U87 cells are transfected with pcDNA3.1-AEG-1/pcDNA3.1 using Lipofectamine™ 2000 for 24 hours and then treated with TGF-β1 (5 ng/ml) or vehicle for another 24 hours. The expression of AEG-1, MAP-LC3 lipidation and BECN1, E-cadherin, vimentin, Snail, Slug and Twist are analyzed by western blot. β-actin is used as internal controls to ascertain equal loading. (**G** and **I**) Representative quantitative data of densitometric analyses. Data represent the mean ± SD of three different experiments. *P < 0.05 and **P < 0.01 compared to vehicle-treated cells or si Control-transfected cells.

Transmission electron microscopy analysis shows the morphological changes in U87 cells induced by AEG-1/MTDH overexpression. Double-membrane vacuolar structures with morphological features of autophagosomes are observed in pcDNA3.1-AEG-1/MTDH-transfected cells compared to pcDNA3.1-transfected cells (Figure 4A). Then, we measure the effect of AEG-1 overexpression on the TGF-β1-triggered the GFP-MAP-LC3 distribution, extensively used as a biomarker for autophagy. The GFP-MAP-LC3 accumulation reaches the most significant accumulation by transiently transfecting U251 and U87 malignant glioma cells with pcDNA3.1-AEG-1/MTDH followed by TGF-β1 treatment for 24 hours (Figure 4B). Figure 4C illustrated the autophagosome-positive rate in each 200 cells. We further study the effect of AEG-1/MTDH overexpression on malignant glioma cells by transiently transfecting U251 and U87 malignant glioma cells with AEG-1/MTDH expression plasmid (pcDNA3.1-AEG-1/MTDH). In pcDNA3.1-treated cells, GFP-LC3 is observed predominantly as a diffuse green fluorescence in the cytoplasm. The autophagosome-positive cells are counted as 1.75 ± 0.35 (U251) and 2.25 ± 0.35 (U87), % of 200 cells. However, characteristic punctate GFP-LC3-positive cells with TGF-β1 treatment are detected as 15.50 ± 1.41 (U251) and 16.25 ± 1.06 (U87), % of 200 cells. AEG-1/MTDH overexpression enhances the autophagosome-positive cells as 45.25 ± 1.06 (U251) and 50.00 ± 0.71 (U87), % of 200 cells (Figure 2D). As shown in Figure 4D and 4E, the overexpression of AEG-1 by pcDNA3.1-AEG-1/MTDH stimulates its downstream cyclin D1 and induces the conversion of the soluble form of MAP-LC3 (LC3-I) to the lipidated and autophagosome-associated form (LC3-II) and the expression of BECN1. The expression of the epithelial marker E-cadherin is strongly reduced when malignant glioma cells are transfected with pcDNA3.1-AEG-1/MTDH. Conversely, the expression of the mesenchymal marker vimentin is enhanced (Figure 4D and 4E). As shown in Figure 4F, 4G, 4H and 4I, the appearance of LC3-II, BECN1 and the mesenchymal marker vimentin, Snail, Slug and Twist are enhanced by pcDNA3.1-AEG-1/MTDH in TGF-β1-stimulated cells, as well as the occurrence of MAP-LC3-positive dots and (Figure 4B and 4C). In contrast, the expression of the epithelial marker E-cadherin is strongly reduced (Figure 4F and 4G). These findings indicate that AEG-1/MTDH overexpression enhances TGF-β1-induced autophagy and EMT in malignant glioma cells. These results demonstrate that AEG-1 stimulates autophagy, an important regulator of cancer survival under metabolic stress, which may underlie its significant EMT-promoting properties.

### AEG-1/MTDH-sensitized autophagy promotes TGF-β1-induced EMT and invasion of malignant glioma cells

**Figure 5:**
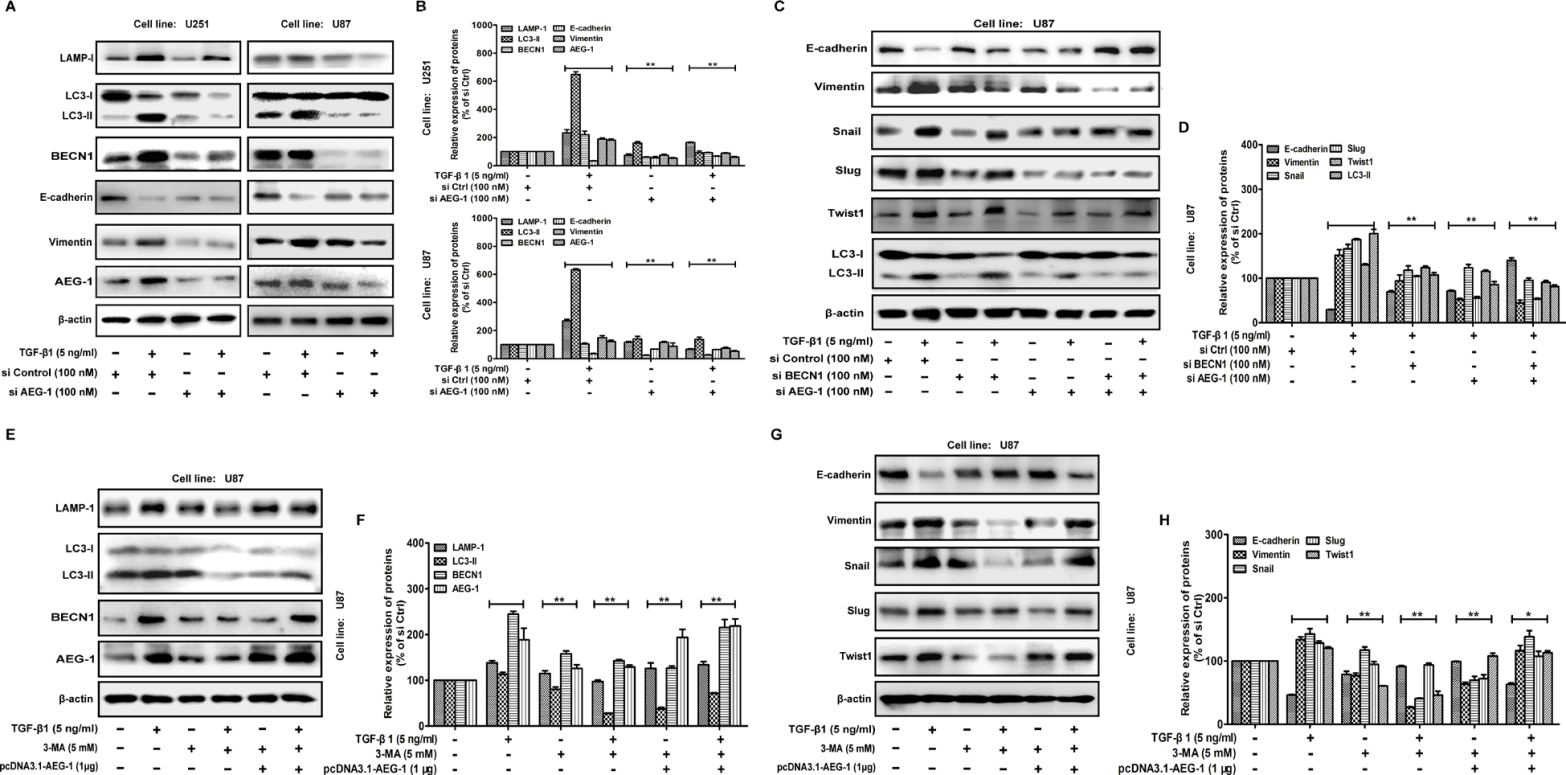
AEG-1 and its downstream autophagy/EMT markers can be rescued by overexpressing AEG-1/TGF-β1 when autophagy is blocked (**A**) Western blot analysis shows the effect of TGF-β1 treatment on U251 and U87 cells transfected with siAEG-1/siControl for 24 hours on MAP-LC3 lipidation and BECN1, LAMP-1, E-cadherin and vimentin expression. β-actin is used as internal controls to ascertain equal loading. (**C**) U87 cells with or without transfection of pcDNA3.1-AEG-1, siAEG-1 and siBECN1 are treated with TGF-β1 (5 ng/ml) for 24 hours, The expression of MAP-LC3 lipidation and E-cadherin, vimentin, Snail, Slug and Twist are analyzed by western blot. β-actin is used as internal controls to ascertain equal loading. (**E** and **G**) Western blot shows the expression of AEG-1 and its downstream autophagy/EMT markers in U87 cells treated with TGF-β1 and/or autophagy inhibitor. The cells are treated with or without TGF-β1 (5 ng/ml) in the presence or absence of 5 mM 3-methyladenine (3-MA) for 24 hours. U87 cells are transfected with pcDNA3.1-AEG-1 in the presence of 5 mM 3-MA for 24 hours. (**B**, **D**, **F** and **H**) Representative quantitative data of densitometric analyses. Data represent the mean ± SD of three different experiments. *P < 0.05 and **P < 0.01 compared to vehicle-treated cells or si Control-transfected cells.

To confirm the contribution of AEG-1 in the TGF-β1-induced autophagy and EMT, we further study the effect of siRNA AEG-1/MTDH on malignant glioma cells. In this study, we use chemically modified AEG-1/MTDH siRNA oligonucleotides for the suppression of direct sites. The inhibition of autophagy by siRNA AEG-1/MTDH weakens autophagy and EMT (Figure 5A and 5B). MAP-LC3 and EMT markers in U87 cells treated by TGF-β1 are significantly inhibited by siRNA AEG-1/MTDH and/or BECN1 followed by TGF-β1 treatment (Figure 5C and 5D). Additionally, we illustrate that both autophagy and EMT markers (vimentin, Snail, Slug and Twist) could be rescued by overexpressing AEG-1/TGF-β1 when autophagy is blocked by autophagy inhibition with 5 mM 3-MA (Figure 5E, 5F, 5G and 5H).

**Figure 6:**
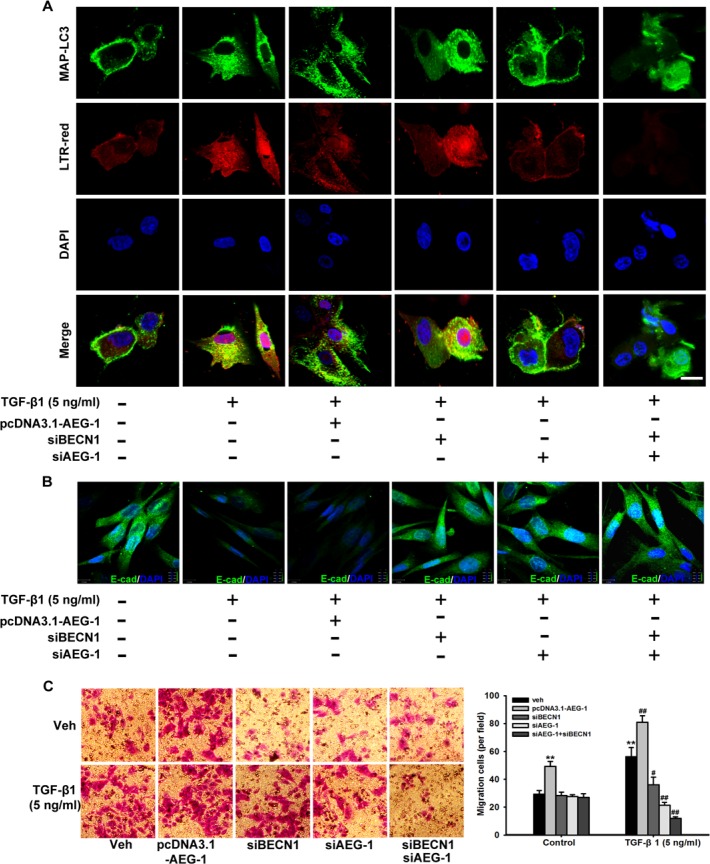
TGF-β1-triggered autophagosome-autolysosome formation activated by AEG-1 and BECN1 drives malignant glioma cell invasion (**A**) Confocal visualization of autophagosome-autolysosome formation is activated by AEG-1 and BECN1 during TGF-β1-induced EMT. U87 cells with or without transfection of pcDNA3.1-AEG-1, siAEG-1 and siBECN1 are treated with TGF-β1 (5 ng/ml) for 24 hours, and confocal fluorescence images of endogenous MAP-LC3 levels and the lysosomal compartments labeled by LTR-red in U87 cells are captured using an FV10-ASW laser scanning confocal microscope [Ver 2.1] (Olympus Corp, MPE FV1000). MAP-LC3 expression (green) is detected using anti-MAP-LC3 polyclonal antibody. Goat anti-rabbit-IgG/FITC is used as a secondary antibody. Cells are simultaneously imaged in the presence of LTR-red to visualize the lysosomal compartment. Nuclei (blue) are labeled by DAPI. Scale bar = 30 μm. (**B**) U87 cells with or without transfection of pcDNA3.1-AEG-1, siAEG-1 and siBECN1 are treated with TGF-β1 (5 ng/ml) for 24 hours and E-cadherin expression is detected by immunofluorescence. Nuclei (blue) were labeled by DAPI. E-cadherin expression (green) is detected using anti-E-cadherin polyclonal antibody. Goat anti-rabbit-IgG/FITC is used as a secondary antibody. Scale bar = 13 μm. Experiments are performed three times; a representative experiment is shown. (**C**) After U87 cells with or without transfection of pcDNA3.1-AEG-1, siAEG-1 and siBECN1 are treated with 50 ng/ml TGF-β1 for 24 hours, the cells are seeded in the upper portion of a Transwell coated with Matrigel. After the cells were incubated for 24 hours, the underside of the membrane is stained with crystal violet solution. Image magnification: 200 ×. The invading cells are counted in five random fields from each treatment. The columns represent the mean ± SD. *P < 0.05 and **P < 0.01 compared to vehicle-treated cells. ^#^*P* < 0.05 and ^##^*P* < 0.01 compared to control cells.

To further confirm that AEG-1 is essential for autophagy, EMT and invasion in response to TGF-β1 treatment, confocal fluorescence microscopy are used to assess the autophagy dynamics. As shown in Figure 6A, malignant glioma cells express the autophagosome marker MAP-LC3 (green fluorescence) fused to its signal in the acidic environment of the autolysosomes (red fluorescence labeled with Lysotracker red [LTR-red]). TGF-β1 treatment of U87 cells reduces basal E-cadherin expression, and siRNA AEG-1/MTDH drastically antagonizes this effect (Figure 6B). Then, we detect the invasion ability of malignant glioma cells following the same treatments to evaluate the relationship between autophagy and invasion induced by TGF-β1 (Figure 6C). MAP-LC3 and LTR-red puncta formation increases significantly in U87 cells transfected with pcDNA3.1-AEG-1 followed by TGF-β1 treatment in Figure 6A. Conversely, the formation of these punctae significantly decreases in U87 cells with siRNA AEG-1 and BECN1 followed by TGF-β1 treatment. Indeed, autophagy inhibition is significantly enhanced by combination interference of AEG-1 and BECN1. Consistent with these data, the reduced basal E-cadherin expression is enhanced by pcDNA3.1-AEG-1 transfection and rescued by combination interference of AEG-1 and BECN1. Transwell invasion assay reveals that migration cells treated by TGF-β1 are enhanced by AEG-1 over-expression from 56.33 ± 6.51 to 81.00 ± 4.58 and decreased by si BECN1 and si AEG-1 to 36.00 ± 5.57 and 21.33 ± 2.08. The combination of si BECN1 and si AEG-1 decreased migration cells to 12.00 ± 1.00 (Figure 6C). These findings suggest that the invasion ability of malignant glioma cells following TGF-β1 treatment is enhanced by autophagy induction and reversed by autophagy inhibition.

### AEG-1/MTDH expression and autophagy and EMT activation are associated with glioblastoma multiforme development and progression

**Figure 7:**
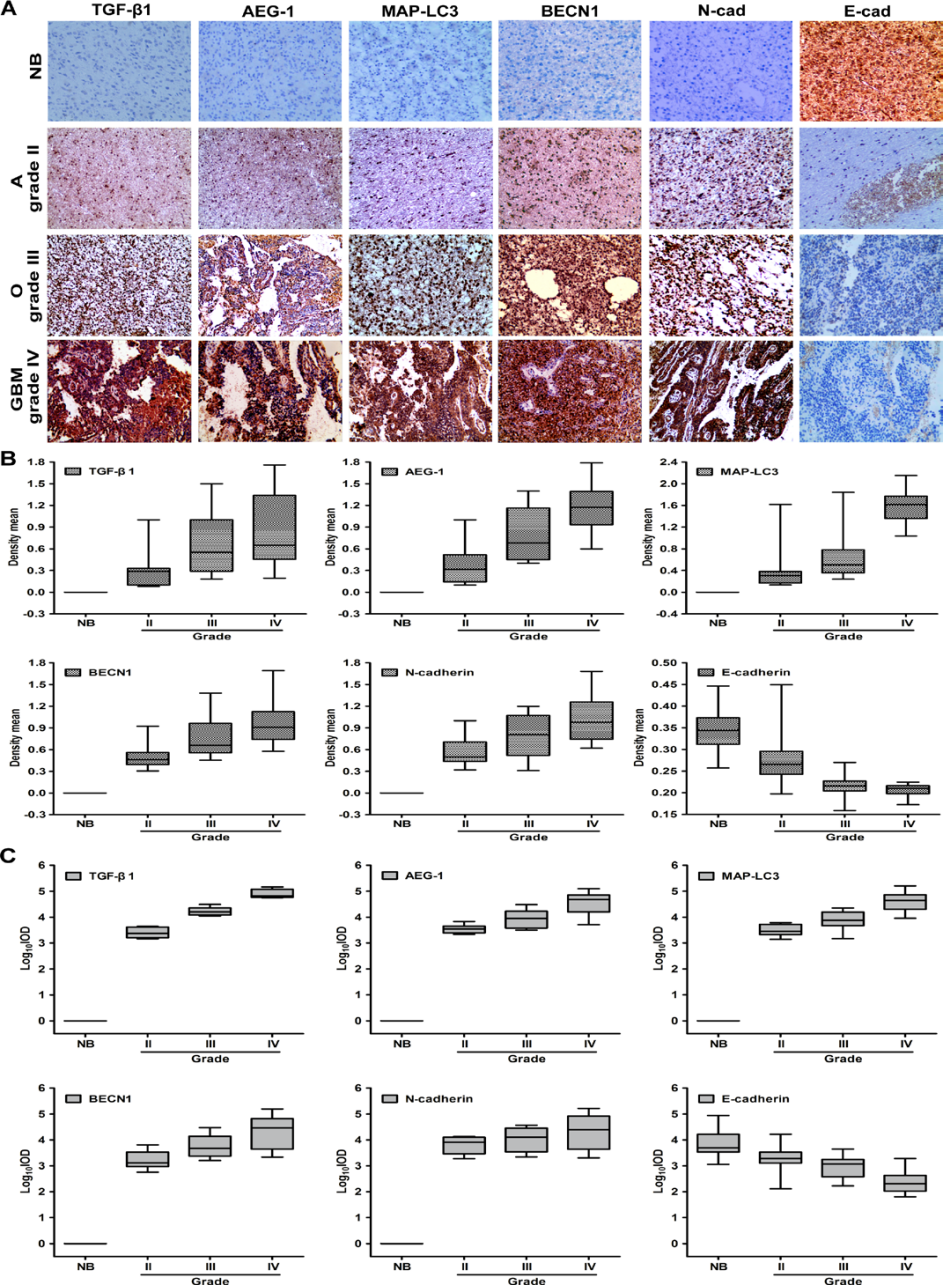
The expression of AEG-1, autophagy hallmarks and EMT is high in diverse malignant glioma tumors (**A**) Representative photomicrographs of TGF-β1, AEG-1, MAP-LC3, BECN1, N-cadherin and E-cadherin expression as determined by IHC staining in 6 samples of anaplastic astrocytoma, 6 samples of oligodendroglioma and oligoastrocytoma, 6 samples of glioblastoma multiforme (GBM), and 6 samples of morphologically normal brain tissue. NB, normal brain; A, anaplastic astrocytoma (grade II); O, oligodendroglioma and oligoastrocytoma (grade III); GBM, glioblastoma multiforme (grade IV). Image magnification: 200×. (**B**) Density mean of TGF-β1, AEG-1, MAP-LC3, BECN1, N-cadherin and E-cadherin expression of diverse malignant gliomas determined by Image Pro-Plus. (**C**) Log10IOD of TGF-β1, AEG-1, MAP-LC3, BECN1, N-cadherin and E-cadherin expression of diverse malignant gliomas determined by Image Pro-Plus. IOD indicates integrated optical density.

We analyze the expression of TGF-β1, AEG-1, hallmarks of autophagy and EMT in patient-derived brain tumor samples to investigate the relationship between AEG-1-sensitized autophagy, EMT and glioblastoma multiforme development and progression. Biopsies are taken from anaplastic astrocytoma (grade II), oligodendroglioma and oligoastrocytoma (grade III), glioblastoma multiforme (grade IV), and normal brain tissue according to the glioma grading classification of the World Health Organization (WHO). We use immunohistochemical (IHC) staining to evaluate TGF-β1, AEG-1, MAP-LC3, BECN1, E-cadherin and N-cadherin expression in formalin-fixed, paraffin-embedded sections of normal brain and brain tumors with glioma grading. As shown in Figure 7A, the normal brain tissue is positive for E-cadherin and negative for TGF-β1, AEG-1, MAP-LC3, BECN1, N-cadherin, except for sparse cytoplasmic staining in a few glia and neurons. Conversely, strong immunostaining for TGF-β1, AEG-1, MAP-LC3, N-cadherin is observed in the glioblastoma multiforme (grade IV) and also in astrocytoma (grade II), oligodendroglioma and oligoastrocytoma (grade III). Additionally, cells with the epithelial phenotype of E-cadherin immunostaining decrease gradually following the malignant glioma grading in tumor samples. Both density mean and IOD are gradually increased from the negative to grade II, to grade III, and to grade IV of TGF-β1, AEG-1, MAP-LC3, BECN1, N-cadherin expression (*P* < 0.001, Figure 7B and 7C, Supplementary Table S1). Both density mean and IOD are gradually decreased from the normal brain to grade II, to grade III, and to grade IV of E-cadherin expression (*P* < 0.001, Figure 7B and 7C, Supplementary Table S1). Although these experiments are only conducted in specimens from a limited number of patients, their results are consistent with the preclinical evidence. Thus, notably high expression of AEG-1 may also trigger autophagy-activated EMT in human malignant glioma.

### SiRNA-AED-1/MTDH reduces tumor volume and induced apoptotic cell death in the rat C6 glioma model

**Figure 8:**
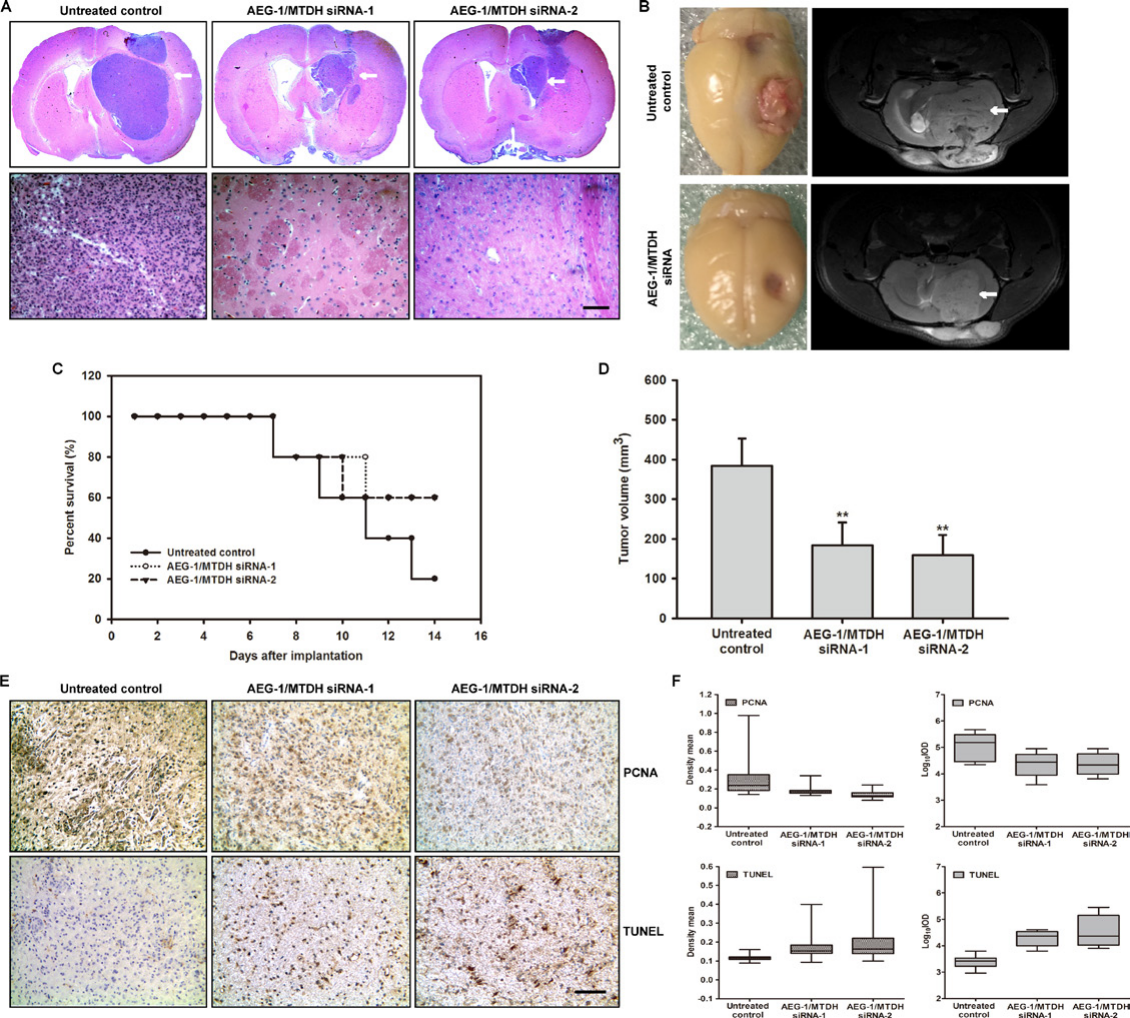
Administration of siRNA-AEG-1/MTDH reduces tumor progression in the rat glioma model (**A**) The magnification of the striatum with a representative part of the tumor by H & E staining. The siRNA-AEG-1/MTDH-treated rats show significantly smaller tumor volumes compared to untreated control rats (Scale bar: 1 mm, upper); The siRNA-AEG-1/MTDH-treated rats show less histopathological changes of the malignant C6 glioma compared to untreated animals (Scale bar: 100 μm, below). (**B**) Frontal brains bearing with C6 glioma and T2-weighted MR images after administration for 14 days. MRI is undertaken on a 7.0 T animal MRI scanner using a 38 mm birdcage rat brain quadrature resonator for radiofrequency transmission and reception as previously described. Images from representative glioma-bearing rats show well established tumor after implantation. Image from siRNA-AEG-1/MTDH administration show the reduced tumor zone (white arrow). (**C**) The percent survival of the glioma-bearing rats. (**D**) C6 glioma volumes in mm3 of AEG-1/MTDH siRNA-treated rats compared with untreated control rats. (**E**) Reduced proliferation and increased apoptosis caused by siRNA-AEG-1/MTDH administration. Ten-micrometer thick cryostat sections are obtained and stained for proliferating cell nuclear antigen (PCNA) or terminal deoxynucleotidyl transferase-mediated deoxyuridine triphosphate nick end labeling (TUNEL), and counterstained with hematoxylin. (**F**) PCNA and TUNEL density mean and Log10IOD determined by Image Pro-Plus (IPP). IOD indicates integrated optical density.

In order to further identify the oncogenic effects of AED-1/MTDH for glioblastoma progression *in vivo*, C6 glioma cells are first transfected with modified siRNA-AED-1/MTDH and implanted into rat striatum. Glioma-bearing rat are intracranially injected with 2 nM modified AED-1/MTDH siRNA-1 and AED-1/MTDH siRNA-2 seven days after initial tumor cell injection once every other day for two weeks (*n* = 5 per group). The H & E staining results reveal that the glioma-bearing rats treated with AED-1/MTDH siRNA exhibit better tumor margins and fewer invasive cells to the contralateral striatum compared with the untreated control rats (Figure 8A). Moreover, the AED-1/MTDH siRNA-treated rats show a dramatic reduction of tumor volume at two weeks of treatment compared with untreated control rats (Figure 8B). The prolonged survival in rats treated with AED-1/MTDH siRNA shows 60%, compared with 20% of untreated control rats (Figure 8C). Untreated control rats exhibit a median tumor volume of 383.92 mm^3^ ± 68.79, whereas AED-1/MTDH siRNA-1 and AED-1/MTDH siRNA-2-treated rats reveal tumor volumes of 184.27 mm^3^ ± 57.16, 159.47 mm^3^ ± 50.60 (Figure 8D).

As shown in Figure 8E, microscopic examination of PCNA-stained tumor sections shows a decrease in PCNA-positive cells in AED-1/MTDH siRNA-treated rats as compared with the untreated controls. Quantitative analyses by density mean and IOD reveal that the proliferation is lower in AED-1/MTDH siRNA-treated rats than that in the untreated controls group (Figure 8F, Supplementary Table S2). The *in vivo* apoptotic response of glioma cells to AED-1/MTDH siRNA-treatment is investigated by TUNEL staining. Microscopic examination of the tumor sections and quantitative evaluation of apoptosis by density mean and IOD shows that compared with the untreated controls, AED-1/MTDH siRNA-treatment increased the number of TUNEL-positive cells (Figure 8E and 8F, Supplementary Table S2).

## DISCUSSION

**Figure 9:**
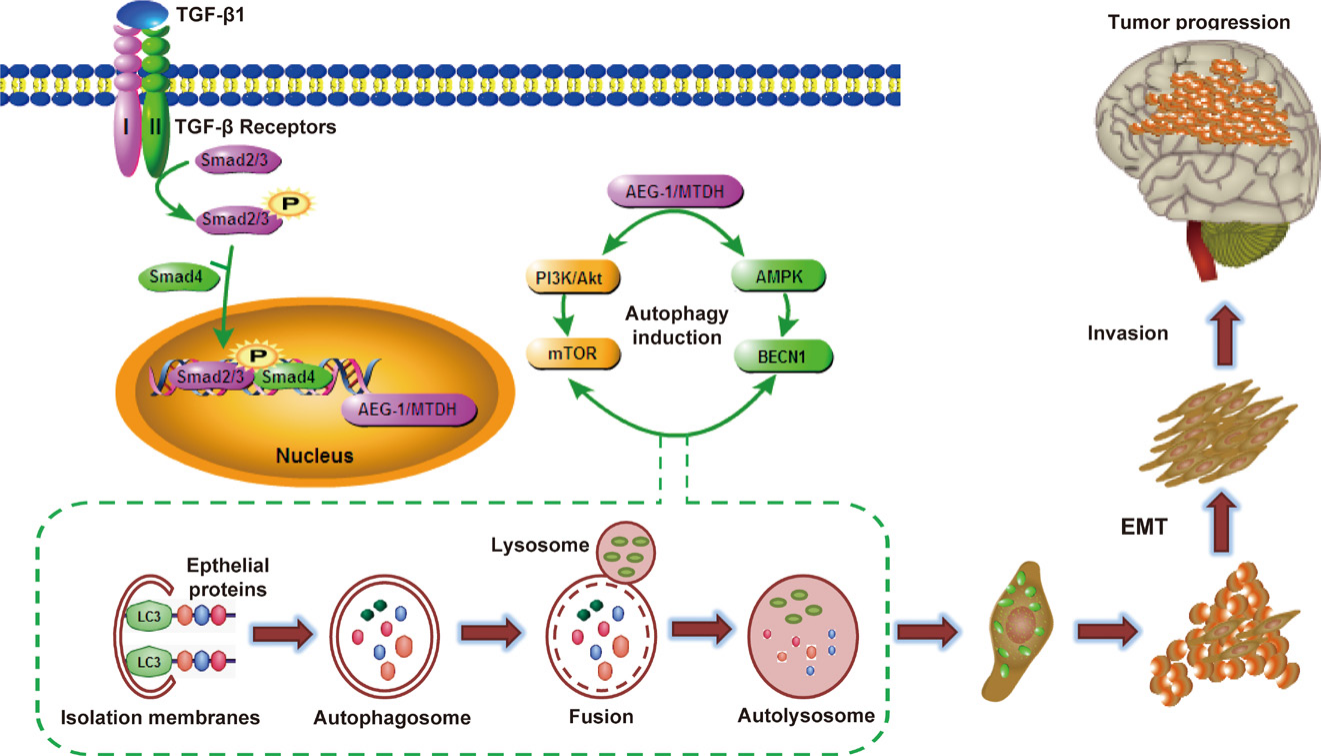
Hypothetical diagram of mechanism for AEG-1/MTDH-activated autophagy enhances human malignant glioma susceptibility to TGF-β1-triggered epithelial-mesenchymal transition During the process of malignant glioma invasion *in vivo*, glioma cells become epithelial-mesenchymal transition. Due to the change of microenvironment and pathways by TGF-β1 activation, the level of AEG-1 increases, causing activation of autophagy. Autophagy promotes EMT via degradation and utilization of intracellular macromolecules and organelles including epithelial markers. This triggers the development of malignant glioma invasion *in vitro* and *in vivo*.

In this study we show that AEG-1/MTDH enhances protective autophagy of malignant glioma cells and promotes TGF-β1-activated EMT. The following evidences support our hypothesis: (a) AEG-1 participates in TGF-β1-triggered EMT and invasion in malignant glioma cells; (b) autophagy is enhanced by AEG-1 over-expression involved in TGF-β1 treatment; (c) autophagy mediates AEG-1-sensitized EMT and invasion; (d) siRNA AEG-1 prevented TGF-β1-induced autophagy and EMT; (e) the coordination of AEG-1 expression and autophagy and EMT activation is associated with the development of anaplastic astrocytoma and glioblastoma; (f) systemically delivered siRNA AEG-1/MTDH reduces tumor volume and induced apoptotic cell death in the rat C6 glioma model. Due to the change of microenvironment and pathways by TGF-β1 activation, the level of AEG-1 increases, causing activation of autophagy. Autophagy promotes EMT via degradation and utilization of intracellular macromolecules and organelles including epithelial markers. This triggers the development of malignant glioma invasion *in vitro* and *in vivo* (Figure 9).

Both the loss of the epithelial phenotype and the acquisition of mesenchymal characteristics are the primary features of EMT [14]. Transformation from a cytoskeleton primarily composed of keratin to a cytoskeleton composed of vimentin can convert epithelial cells into spindle-like cells. This phenotypic transformation allows tumor cells to escape from cell adhesion, thereby becoming more invasive [22]. The reduced expression of cell adhesion-related molecules such as E-cadherin and increased expression of N-cadherin during EMT can result in reduced intercellular adhesion of epithelial cells. EMT and tumor progression are complex biological processes, and the exact mechanism underlying the regulation of EMT has not been elucidated.

The typical progression of autophagy includes the following aspects [11, 12, 23, 24]: activated double membranes are shed from the rough endoplasmic reticulum region without ribosomes and wrap cytoplasm, degraded organelles and proteins into autophagosomes (initial autophagic vacuoles, AVi); lysosome fusion with the autophagosome results in the formation of an autolysosome and degradation of its contents [25, 26]. We deepen our understanding of the morphological characteristics, molecular mechanisms and physiological functions involved in this process. During the early stages of autophagy induction, signal transduction through the Vps34 (Class III PI3k)/Beclin 1 (BECN1) complex is essential for the formation of autophagic vacuoles [27]. Microtubule-associated protein LC3 (MAP-LC3), a mammalian homolog of the yeast Atg8 gene, is essential for autophagosome formation. LC3-II can promote the extension of the autophagosome with its amalgamation properties. LC3-II is tightly attached to the initial autophagocytic vacuole and autophagosome membrane and thus becomes a standard symbol of autophagy measurement [28].

AEG-1 is considered as a cancer promoter and stimulator of apoptosis [29], autophagy [10], invasion, metastasis, angiogenesis [30] and drug resistance. AEG-1 promotes oncogene expression through activating the PI3k-Akt, NF-κB and Wnt signaling pathways [31, 32]. Recently, AEG-1 has been reported to induce protective autophagy; however, the relationship between AEG-1-activated autophagy and invasion of glioma cells remains elusive [10]. We hypothesized that reducing AEG-1 expression and thereby inhibiting protective autophagy may be a feasible and effective method for treating cancer. Recent studies have shown that the protective mechanism of AEG-1 in serum-starved cells is related to AEG-1-activated autophagy. Moreover, the inhibition of AEG-1-induced autophagy leads to the acceleration of serum starvation-induced cell death [10]. However, the detailed contribution of AEG-1 expression and autophagy induction to malignant glioma is not clear. Our study is the first to reveal a unique perspective that AEG-1 promotes the invasion and progression of malignant glioma cells by promoting TGF-β1-triggered EMT via the induction of autophagy. Autophagic flux is activated within 3 hours and accompanied by the up-regulation of AEG-1 in malignant glioma cells following TGF-β1 (5 ng/ml) treatment for 1, 3, 6, 12 and 24 hours. Simultaneously, the expression of the oncogene cyclin D1 and EMT markers increased along with AEG-1, suggesting that this process may promote tumor progression. Autophagy inhibition by siBECN1 and siAEG-1 pretreatment reversed the expression levels of the above markers. AEG-1 could be a potential target for a variety of tumor therapies.

TGF-β1 is secreted and expressed at high levels in glioma and may be the primary factor that promotes the progression of malignant glioma. Our research focuses on the model of TGF-β1-induced malignant glioma cell invasion and EMT *in vitro*. In summary, our results show that the up-regulation of AEG-1 by TGF-β1 activates protective autophagy and promotes EMT to promote malignant glioma cell proliferation, invasion and metastasis. Moreover, down-regulating the AEG-1 level in malignant glioma by pharmacological inhibition or genetic interference may contribute to the prevention and reduction of glioma invasion and progression. Our present studies are focusing on the relationship between autophagy and EMT. The selective autophagy receptor and signaling adaptor sequestosome-1 (SQSTM1/p62) is a multifunctional protein. The presence of an LC3-interacting region (LIR) enables SQSTM1/p62 to bind to the autophagy protein LC3, and the ubiquitin-associated domain (UBA) binds to ubiquitin to mediate the selective degradation of ubiquitinated cargo by autophagy [33]. SQSTM1/p62 contains various ubiquitin-associated domains that facilitate interaction with many signaling activators, including AEG-1/MTDH and TGF-β/Smad signaling [34]. We are trying to elucidate the mechanism how SQSTM1/p62 increases AEG-1 and junctional proteins through Epithelial-Mesenchymal Transition factors in TGF-β1-treated glioma cells. The systematic work is in progress and many more experiments are needed to support our hypothesis.

## MATERIALS AND METHODS

### Cell lines, cell culture and human tissue samples

The human malignant glioma cell lines U251, U87 and rat glioma cell line C6 are routinely obtained from Cell Bank of Shanghai Institute of Biochemistry and Cell Biology, Chinese Academy of Sciences (Shanghai, China). The U251 and U87 cells are maintained in DMEM (Gibco, Grand Island, NY) medium supplemented with 10% fetal bovine serum (Gibco, Grand Island, NY) in a humidified atmosphere at 37°C with 5% CO_2_. Only cells from generations 3 to 8 are used for the experiments. The C6 cells are cultured in F12K (Gibco, Grand Island, NY) medium supplemented with 10% fetal calf serum (Gibco, Grand Island, NY), 100 U/ml penicillin and 100 U/ml streptomycin at 37°C in a 5% CO_2_ atmosphere. Only the second generation of cells after recovery from liquid nitrogen is used in the following experiments.

The archival frozen human tissue specimens used in this study consist of 6 samples of astrocytoma, 6 samples of oligodendroglioma and oligoastrocytoma, 6 samples of glioblastoma multiforme (GBM), and 6 samples of morphologically normal brain tissue that are surgically resected at the First Affiliated Hospital of Nanjing Medical University between Jan 2014 and Dec 2014. Further classification of glioma grading is based on the malignant degree of astrocytoma according to the standard of the WHO, including atypical characteristics, mitotic index, endothelial cell proliferation and necrotic degree.

### Animals

Adult male Spraque-Dawley rats (Charles River Laboratories, Sulzfeld, Germany), weighting 250–300 g are raised in Specific pathogen Free (SPF) grade animal laboratory. Animals are housed in groups of two under standard conditions at a temperature of 22°C ± 1 and a 12 h-12 h light/dark cycle starting at 7:00 AM with free access to food and water. All experiments are carried out according to the National Institutes of Health Guide for the Care and Use of Laboratory Animals (publication no. 85–23, revised 1985) and approved by IACUC (Institutional Animal Care and Use Committee of Nanjing Medical University, Ethical no.14030134).

### Orthotopic glioma model

The rats are divided into three groups. The negative control group is implanted with untreated control C6 cells. The other two group is implanted with AEG-1/MTDH siRNA-1 and AEG-1/MTDH siRNA-2-transfected C6 cells. Before implantation, 85–90% confluent C6 cells are trypsinized, rinsed with F12K+10% fetal calf serum, and centrifuged at 1000 rpm for 4 min. The cell pellet is resuspended in F12K and placed on ice. Concentration of viable cells is adjusted to 1 × 10^8^ cells/ml of F12K. Each rat is anesthetized by a peritoneal injection with 0.4 ml/100 g of 1% pentobarbital sodium solution and placed in a stereotactic frame (RWD Life Science, Shenzhen, China). After shaving and disinfection of the skin, a sagittal incision is made to expose the skull, followed by a burr hole 1 mm anterior and 3 mm lateral relative to the bregma using a small drill. Cell suspension is injected into a depth of 6 mm from the skull surface, using a 2 μl Hamilton (#2701) syringe (Reno, NV, USA) with a 26s-gauge needle mounted on a stereotactic holder. On completion of the injection, the needle is left in place for 5 min and withdrawn slowly. The scalp incision is then closed with surgical sutures. The animals are injected intramuscularly with 0.1 ml/rat of 80 U/ml benzylpenicillin sodium solutions for prevention of infection and returned to their home cages. [35] 2 nM AEG-1/MTDH siRNA-1 and AEG-1/MTDH siRNA-2 are intratumorally administered once every other day using a stereotactic technique on day 7 after tumor implantation. The tumor volume is calculated as: (square root of maximal tumor cross-sectional area)^3^.

### Magnetic resonance imaging (MRI)

MRI is used in the rat C6 glioma model for confirmation of the volume of tumor on day 14 after implantation. MRI is undertaken on a 7.0 T animal MRI scanner (70/16 PharmaScan, Bruker Biospin GmbH, Germany) using a 38 mm birdcage rat brain quadrature resonator for radiofrequency transmission and reception. Briefly, rats are anesthetized using inhaled isoflurane/O_2_ (3% for induction and 1.5–2% for maintenance). During the MRI scan, the rats are prostrated on a custom made holder to minimize head motion while respiration is maintained at a rate of 50 breaths/min. Scout T_2_-weighted imaging (T_2_WI) in three planes with a fast spin echo pulse sequence is first acquired to control rat head positioning. Next, a coronal T_2_WI scan is acquired using a rapid-acquisition relaxation-enhancement pulse sequence with the following parameters: field of view = 3 × 3 cm, matrix size = 256 × 256, repetition time = 2500 ms, echo time = 33 ms, slice thickness = 1.0 mm, slice gap = 1.0 mm, and acquisition time = 1 min 20 s. [35]

### Histology, immunohistochemistry assay

Animals are deeply anesthetized and perfused transcardially with 4% paraformaldehyde in phosphate-buffered saline (PBS). Brains are removed from the skulls and post-fixed overnight at 4°C in 4% paraformaldehyde. Next day, the brains are transferred to 30% sucrose in PBS solution for 48 h at 4°C. Coronal sections with a thickness of 10 mm are cut using a cryostat microtome (Leica CM1900, Germany). Hematoxylin and eosin (H & E) staining is used to visualize the tumor area and tumor necrosis. For evaluating cell proliferation, immunostaining for proliferating cell nuclear antigen (PCNA) is used. Antibody against PCNA (Abcam) is diluted 1: 300 in blocking solution containing 0.3% Triton X-100 and incubated overnight at 4°C. After being washed in PBS, the sections are incubated with a biotinylated secondary antibody for 2 h. They are washed and further incubated with a streptavidin-biotin-peroxidase complex (Vector Laboratories). Apoptosis is examined using the terminal deoxynucleotidyl transferase-mediated deoxyuridine triphosphate nick end labeling (TUNEL) method (*In Situ* Cell Death Detection Kit; Roche Molecular Biochemicals) according to the manufacturer's instructions. Then quantification of the expression of PCNA and TUNEL is measured using professional Image-Pro-Plus 6.0 (IPP) [three parameters: area, density mean, and integrated optical density (IOD)].

### Reagents

Recombinant human transforming growth factor β1 (TGF-β1, cat. #AF-100-21C, PeproTech, Rocky Hill, USA) is applied to induce malignant glioma cells to undergo EMT. This reagent is dissolved in triple-distilled water at a concentration 100 μg/ml and stored at −80°C. A stock solution is diluted to the appropriate concentrations with growth medium immediately before use. Lipofectamine^™^ 2000 transfection reagent is purchased from Life Technologies Co. (Invitrogen, Carlsbad, CA, USA). 3-methyladenine (3-MA, Sigma-Aldrich, Inc., St. Louis, MO) is diluted to 5 mM. Cholesterol-conjugated siRNA with chemical modification of 2′-O-Me for *in vivo* RNA interference and its negative control are from GenePharma, Inc. (Pudong, Shanghai)

### Antibodies

Primary antibodies directed against AEG-1/lyric/metadherin (CST-9596), MAP-LC3 (CST-12741), TGF-β1 (CST-3711), LAMP-1 (CST-9091) and BECN1 (CST-4122) are purchased from Cell Signaling Technology, Inc. (Beverly, MA). Primary antibodies directed against E-cadherin (BS-1097), N-cadherin (BS-2224), vimentin (BS-1855), mTOR (BS-3611) and p-mTOR (BS-4706) are purchased from Bioworld Technology Co., Ltd. Primary antibody directed against β-actin (BM0627) is purchased from Boster Biological Technology, Ltd. Primary antibodies directed against AEG-1/MTDH (13860-1-AP) Snail (26183-1-AP), Twist1 (25465-1-AP), Slug (12129-1-AP) are purchased from Proteintech Technology, Inc. (Wuhan, China). Primary antibodies against Smad2/3 (sc-6202), p-Smad2/3 (Ser 423/425) (sc-11769) and Smad2/3 siRNA (sc-37238) are purchased from Santa Cruz Technology, Ltd. (Santa Cruz, CA). The secondary antibodies used are as follows: (1) anti-mouse IgG: IRDye^™^ 800-conjugated anti-mouse IgG (Rockland, Inc., Philadelphia, PA); (2) anti-rabbit IgG: Alexa Fluor 680 goat anti-rabbit IgG (Invitrogen, Carlsbad, CA); (3) anti-goat IgG: Alexa Fluor 680 rabbit anti-goat IgG (Invitrogen, Carlsbad, CA); (4) goat anti-mouse IgG HRP (SC-2302, Santa Cruz Biotechnology, Inc., Texas, USA); (5) goat anti-rabbit IgG HRP (SC-2030, Santa Cruz Biotechnology, Inc., Texas, USA).

### Transient transfection

The pcDNA3.1-GFP-LC3 fusion plasmid is kindly provided by Dr. Rong Mu (Peking Union Medical College). The pcDNA3.1-AEG-1/MTDH plasmid is kindly provided by Dr. Kunmei Liu (Ningxia Medical University). For transfection, the cells are trypsinized and seeded in 6-well plates at a density of 5000 cells/well. Plasmids are introduced at 2 μg/well or 1 μg/well into the cells using Lipofectamine^™^ 2000 (Invitrogen, Carlsbad, CA) according to the manufacturer's recommendations. The expression vector is transfected 24 hours before treatment with TGF-β1.

### siRNA interference

The human AEG-1/MTDH siRNA oligonucleotides (Sequence 1: sense 5′-GCAGCAAGGCAGTCTTTAAG T-3′, antisense 5′-ACTTAAAGACTGCCTTGCTGC-3′; Sequence 2: sense 5′-GUUACCACCGAGCAACUUAdT dT-3′, antisense 5′-UAAGUUGCUCGGUGGUAACdT dT-3′), Beclin-1 (BECN1) siRNA oligonucleotides (Sequence 1: sense 5′-AAGAUUGAAGACACAGGAG GC-3′, antisense 5′-GCCUCCUGUGUCUUCAAUCU U-3′; Sequence 2: sense 5′-CAGUUUGGCACAAUCAA UA-3′, antisense 5′-UAUUGAUUGUGCCAAACUG-3′) and the rat AEG-1/MTDH siRNA oligonucleotides (Sequence 1: sense 5′-CAACAGCGUAAACGUGAUA TT-3′, antisense 5′-UAUCACGUUUACGCUGUUGTT-3′; Sequence 2: sense 5′-CACCGAGCAACUUACAACU TT-3′, antisense 5′-AGUUGUAAGUUGCUCGGUG TT-3′; Sequence 3: sense 5′-GGGAAGGAAUUGGAGU GAUTT-3′, antisense 5′-AUCACUCCAAUUCCUUCCC TT-3′) are synthesized by GenePharma, Inc. (Pudong, Shanghai). A universal negative control siRNA is used. We perform siRNA transfection using Lipofectamine^™^ 2000 (Invitrogen, Carlsbad, CA) according to the manufacturer's instructions. Briefly, the cells that reached 50% confluence are transfected with serum-free DMEM medium containing 50 nmol/l scrambled siRNA, AEG-1, or BECN1 siRNA for 6 hours, followed by recovery in medium containing serum and other special treatments as necessary.

### Wound healing assay

The wound healing assay is one of the earliest developed methods to study directional cell migration *in vitro* [36]. Malignant glioma cells are seeded in a 6-well plate and allowed to attach overnight to 80% confluence. Subsequently, cell monolayers are wounded using white pipette tips and washed twice with PBS to remove floating cells. Then, the cells are incubated in DMEM medium with TGF-β1 (5 ng/ml) for up to 24 hours. The cells migrate into the wound surface, and the number of migrating cells is determined using an inverted microscope at 12 and 24 hours. We have analyzed five randomly chosen fields for each well.

### Transwell invasion assay

The invasion behavior of U251 and U87 cells is determined using 24-well Millicell Hanging Cell Culture inserts with 8 mm PET membranes (Millipore, Bedford, Massachusetts, USA) as described previously [37]. Briefly, after the cells are treated with TGF-β1 (5 ng/ml) for 12 and 24 hours, 5.0 × 10^4^ U251 and U87 cells in 200 μl serum-free DMEM medium are plated onto BD BioCoat^™^ Matrigel^™^ Invasion Chambers (8 μM pore size polycarbonate filters; BD Biosciences), while complete medium containing 10% FBS is added to the lower chamber. After processing the invasion chambers for 24 hours (37°C, 5% CO_2_) in accordance with the manufacturer's protocol, the non-invading cells are removed with a cotton swab; the invading cells are fixed in 100% methanol and then stained with crystal violet solution. The invading cells on the lower surface of the membrane filter are counted microscopically. The data are presented as the average number of cells attached to the bottom surface from five randomly chosen fields.

### Cell lysate extraction and western blot analyses

Total cellular proteins are extracted with lysis buffer (50 mmol/l Tris [pH 7.4], 150 mmol/l NaCl, 1% Triton X-100, 1% deoxycholic phenylmethylsulfonyl fluoride, 1 mg/ml aprotinin, 5.0 mm sodium pyrophosphate, 1.0 g/ml leupeptin, 0.1 mm phenylmethylsulfonyl fluoride, and 1 mm/l DTT). Protease inhibitors are added immediately before use. The cells are harvested at the end of treatment and extracted for western blot analysis as described previously [38].

### IF and confocal fluorescence microscopy

Cells treated for the indicated times are fixed in 4% paraformaldehyde in PBS at 1 hour intervals, permeabilized with 0.5% Triton X-100, and blocked with 2% BSA for 30 minutes. The cells are incubated with primary antibody (diluted 1:50) directed against MAP-LC3 or E-cadherin overnight at 4°C. Lysosomal-rich/acidic compartments are visualized with LTR-red (Beyotime, C1046) and incubated at a final concentration of 50 nM for 1 hour. The nuclei are stained with 4′,6-diamidino-2-phenylindole (DAPI, Sigma-Aldrich, St. Louis, MO) for 10 minutes before imaging. An FV10-ASW laser scanning confocal microscope [Ver 2.1] (Olympus Corp, MPE FV1000) is used for co-localization analysis [38].

### Transmission electron microscopy

U87 cells are pretreated with TGF-β1 (5 ng/ml) for 24 hours or transfected with pcDNA3.1/pcDNA3. 1-AEG-1/MTDH for 24 hours. The cells are fixed immediately in 1% glutaraldehyde and post-fixed in 2% osmium tetroxide. Then, the cell pellets or sections are embedded in epon resin. Representative areas are chosen for ultrathin sectioning and viewed using a FEI Tecnai G2 Spirit Bio TWIN transmission electron microscope (FEI Co., Netherlands) at an acceleration voltage of 120 kV.

### IHC analysis

IHC staining of tumor tissue using AEG-1/MTDH-specific rabbit polyclonal antibody (#13860-1-AP), MAP-LC3 (CST-12741), TGF-β1 (CST-3711), E-cadherin (BS-1097) and N-cadherin (BS-2224) antibodies is performed as described previously [39]. Briefly, human tumor samples are subjected to deparaffinization, rehydration, and antigen retrieval before the staining procedures are performed. The tissue slides are blocked with 2.5% normal horse serum for 10 minutes. Then, tissue slides are incubated with AEG-1/MTDH-specific rabbit polyclonal antibody, MAP-LC3, TGF-β1, E-cadherin and N-cadherin antibody (dilution 1:50) overnight at 4°C. After the tissue slides are washed, they are incubated with anti-mouse IgG HRP and anti-rabbit IgG HRP secondary antibody for 10 minutes. The slides are stained with 3, 3′-diaminobenzidine (DAB) (Vector Laboratories), counterstained with hematoxylin (Vector Laboratories), dehydrated, treated with xylene, and mounted. All slides are examined and representative pictures are taken using an Olympus B × 41 microscope (Olympus America, Melville, NY) [40].

### Statistical analysis

All data are expressed as mean ± SD and statistically compared by one-way ANOVA with Dunnett's test and post-hoc tests are undertaken using the GraphPad Prism 5.0 software. The details of each statistical analysis used are presented in the figure legends. Significance is indicated as *P < 0.05, ^#^*P* < 0.05 and **P < 0.01, ^##^*P* < 0.01.

## SUPPLEMENTARY MATERIALS FIGURE AND TABLES



## Abbreviations

3-MA: 3-methyladenine
AEG-1: astrocyte elevated gene 1
AVs: autophagic vacuoles
BECN1: Beclin 1
cath D: cathepsin D
MTDH: Metadherin
EMT: epithelial mesenchymal transition
GBM: glioblastoma multiforme
GFP: green fluorescent protein
IF: immunofluorescence
IHC: immunohistochemistry
LAMP-1: lysosome-associated membrane protein
LTR-red: lysotracker red
MAP-LC3: microtubule associated protein 1 light chain 3
MRI: Magnetic resonance imaging
NB: normal brain
PCNA: proliferating cell nuclear antigen
PI3K: phosphatidylinositol 3-kinase
SQSTM1/p62: signaling adaptor sequestosome-1
TEM: transmission electron microscopy
TGF-β1: transforming growth factor-β1
TUNEL: transferase-mediated deoxyuridine triphosphate-biotin nick end labeling
UBA: ubiquitin-associated domain.
