# Supplementary material for: AEG-1/MTDH-activated autophagy enhances human malignant glioma susceptibility to TGF-β1-triggered epithelial-mesenchymal transition
Source: Oncotarget. 2016 Feb 20;7(11):13122–38. doi: 10.18632/oncotarget.7536 (PMC4914346; doi:10.18632/oncotarget.7536)
Supplement: Supplementary file 1 [file oncotarget-07-13122-s001.pdf]

# AEG-1/MTDH-activated autophagy enhances human malignant glioma susceptibility to TGF-β1-triggered epithelial-mesenchymal transition

## Supplementary Materials

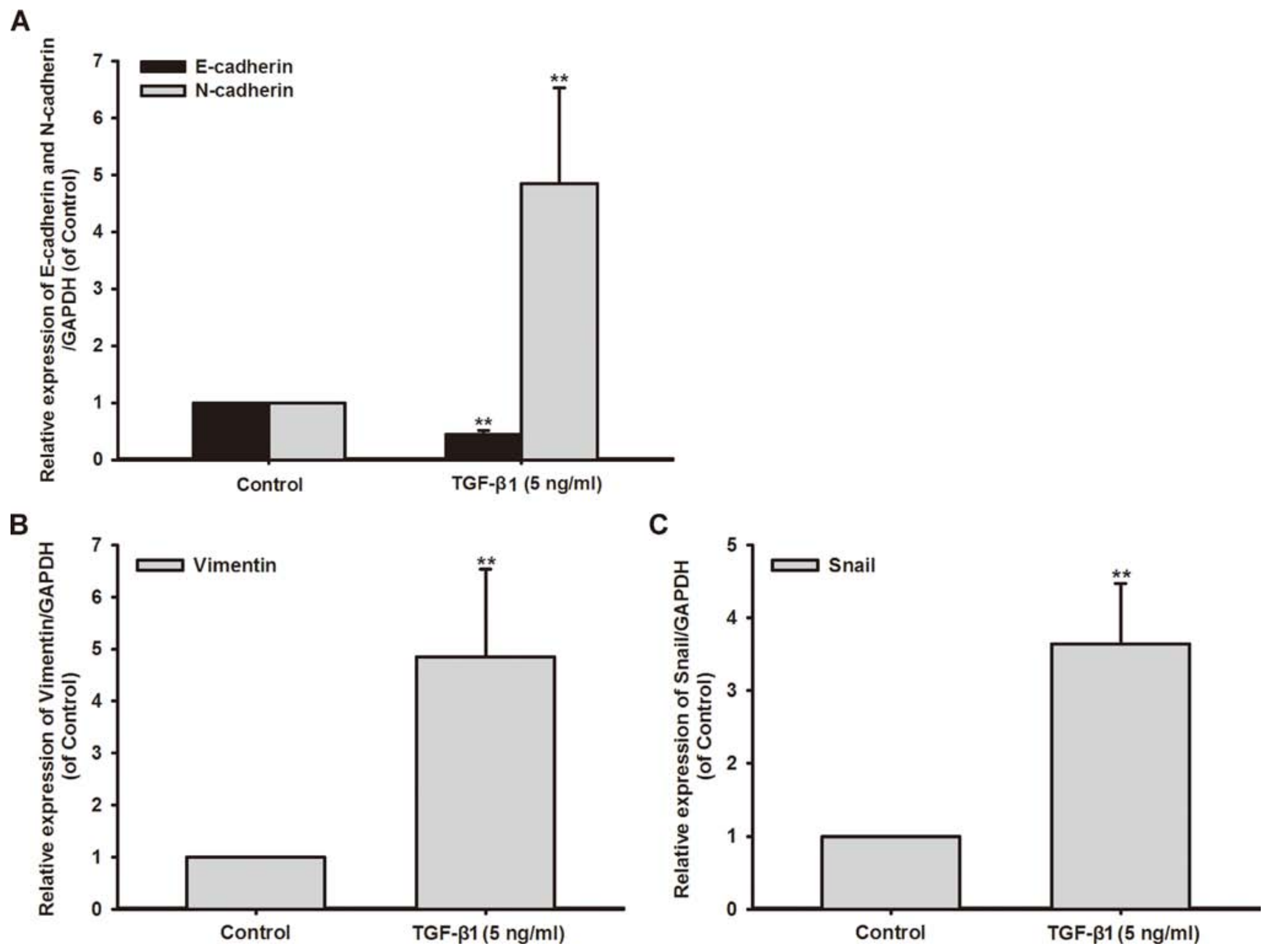


**Supplementary Figure S1: Figure S1: Relative gene expression of E-cadherin, N-cadherin, Vimentin and Snail.** The cells are treated with TGF-β1 (5 ng/ml) and vehicle for 24 hours. Total RNA was extracted using Trizol reagent (Invitrogen, USA). Reverse transcription-PCR was performed with M-MLV (Promega, USA) following standard protocols. For the TaqMan-based real-time reverse transcription-polymerase chain reaction (RT-PCR) assays, the ABI 7900 HT Sequence Detection system (Applied Biosystem, Foster City, CA) was used. For quantitative PCR of mRNA, the E-cadherin, N-cadherin, Vimentin, Snail, and GAPDH primers were purchased from Invitrogen (Life technology). EzOmics SYBR qPCR kit was purchased from Biomics. Amplification procedure was 94°C for 5 min, followed by 30 cycles at 94°C for 30 s, 61°C for 45 s, finally 72°C for 10 min.

**Supplementary Table S1: Expressions of AEG-1, autophagy hallmarks and EMT in relation to malignant glioma tumors grades**

| Markers | Glioma Grade | Area | | Density Mean | | $Log_{10}$IOD | |
|---|---|---|---|---|---|---|---|
| | | Mean ± SD | *P* | Mean ± SD | *P* | Mean ± SD | *P* |
| TGF-β1 | | | $P < 0.01$ | | $P < 0.01$ | | $P < 0.05$ |
| | Grade II | 2.823 ± 2.142 | a | 0.253 ± 0.153 | a | 3.391 ± 0.183 | a |
| | Grade III | 10.985 ± 7.806 | b | 0.660 ± 0.419 | b | 4.217 ± 0.155 | b |
| | Grade IV | 277.679 ± 16.117 | c | 0.822 ± 0.561 | c | 4.892 ± 0.166 | c |
| AEG-1 | | | $P < 0.01$ | | $P < 0.01$ | | $P < 0.05$ |
| | Grade II | 5.153 ± 2.939 | a | 0.361 ± 0.222 | a | 3.542 ± 0.161 | a |
| | Grade III | 13.598 ± 6.446 | b | 0.806 ± 0.357 | b | 3.938 ± 0.360 | b |
| | Grade IV | 53.306 ± 11.475 | c | 1.165 ± 0.294 | c | 4.561 ± 0.462 | c |
| MAP-LC3 | | | $P < 0.01$ | | $P < 0.01$ | | $P < 0.05$ |
| | Grade II | 3.419 ± 7.572 | a | 0.332 ± 0.210 | a | 3.509 ± 0.232 | a |
| | Grade III | 11.555 ± 4.959 | b | 0.616 ± 0.320 | b | 3.867 ± 0.397 | a |
| | Grade IV | 21.091 ± 20.355 | c | 1.567 ± 0.255 | c | 4.602 ± 0.412 | b |
| BECN1 | | | $P < 0.01$ | | $P < 0.01$ | | $P < 0.05$ |
| | Grade II | 12.907 ± 8.131 | a | 0.486 ± 0.114 | a | 3.206 ± 0.358 | a |
| | Grade III | 24.624 ± 13.343 | b | 0.757 ± 0.235 | b | 3.748 ± 0.463 | b |
| | Grade IV | 66.314 ± 11.269 | c | 0.949 ± 0.251 | c | 4.264 ± 0.707 | c |
| N-cadherin | | | $P < 0.01$ | | $P < 0.01$ | | $P < 0.05$ |
| | Grade II | 13.893 ± 7.093 | a | 0.569 ± 0.186 | a | 3.808 ± 0.350 | a |
| | Grade III | 24.780 ± 9.940 | b | 0.773 ± 0.299 | b | 4.019 ± 0.486 | a |
| | Grade IV | 219.517 ± 23.708 | c | 1.013 ± 0.293 | c | 4.311 ± 0.719 | b |
| E-cadherin | | | $P < 0.05$ | | $P < 0.05$ | | $P < 0.05$ |
| | NB | 42.344 ± 8.257 | a | 0.345 ± 0.041 | a | 3.871 ± 0.546 | a |
| | Grade II | 24.132 ± 8.653 | b | 0.276 ± 0.047 | b | 3.245 ± 0.626 | b |
| | Grade III | 9.051 ± 4.127 | c | 0.215 ± 0.018 | c | 3.007 ± 0.468 | b |
| | Grade IV | 8.200 ± 4.262 | c | 0.207 ± 0.012 | c | 2.397 ± 0.494 | c |

Values are expressed as mean (standard deviation). IOD indicates integrated optical density; All data were expressed as mean ± SD and statistically compared by one-way ANOVA with Dunnett's test and post-hoc tests were undertaken using the GraphPad Prism 5.0 software. ($P < 0.01$, $P < 0.05$, a > b > c > d).

**Supplementary Table S2: Expressions of PCNA and TUNEL in relation to rat C6 glioma tumors**

| Markers | Glioma Grade | Area | | Density Mean | | Log10IOD | |
|---|---|---|---|---|---|---|---|
| | | Mean ± SD | *P* | Mean ± SD | *P* | Mean ± SD | *P* |
| PCNA | | | $P < 0.01$ | | $P < 0.05$ | | $P < 0.05$ |
| | Control | 117.193 ± 29.038 | a | 0.268 ± 0.102 | a | 5.047 ± 0.526 | a |
| | SiRNA-1 | 80.799 ± 23.102 | b | 0.255 ± 0.014 | b | 4.359 ± 0.485 | b |
| | SiRNA-2 | 67.324 ± 17.276 | c | 0.141 ± 0.040 | c | 4.366 ± 0.424 | c |
| TUNEL | | | $P < 0.01$ | | $P < 0.05$ | | $P < 0.05$ |
| | Control | 8.019 ± 3.513 | a | 0.115 ± 0.010 | a | 3.394 ± 0.268 | a |
| | SiRNA-1 | 17.852 ± 4.195 | c | 0.173 ± 0.050 | c | 4.286 ± 0.312 | c |
| | SiRNA-2 | 18.480 ± 7.339 | c | 0.188 ± 0.070 | c | 4.543 ± 0.617 | c |

Values are expressed as mean (standard deviation). IOD indicates integrated optical density; All data were expressed as mean ± SD and statistically compared by one-way ANOVA with Dunnett's test and post-hoc tests were undertaken using the GraphPad Prism 5.0 software. ($P < 0.01$, $P < 0.05$, a > b > c).
